# Recombinant *Bacillus subtilis* Improves Growth and Protein Utilization and Regulates PI3K/AKT/TOR Pathway-Related Genes in Chinese Mitten Crab (*Eriocheir sinensis*)

**DOI:** 10.3390/ani15121732

**Published:** 2025-06-12

**Authors:** Yongmin Li, Shengyu Gao, Wenbin Liu, Xiangfei Li, Guangzhen Jiang, Chaofan He

**Affiliations:** 1 Rural Revitalization Collaborative Technology Service Center of Anhui Province, Fuyang Normal University, Fuyang 236041, China; lyminron@163.com (Y.L.); gsy1596602032@163.com (S.G.); 2 Key Laboratory of Aquatic Nutrition and Feed Science of Jiangsu Province, College of Animal Science and Technology, Nanjing Agricultural University, Nanjing 210095, China; wbliu@njau.edu.cn (W.L.); xfli@njau.edu.cn (X.L.); jianggz@njau.edu.cn (G.J.)

**Keywords:** P4’ peptide, recombinant *Bacillus subtilis*, hepatopancreatic gene expression, *Eriocheir sinensis*

## Abstract

The inclusion of suitable quantities of plant protein hydrolysates in feed enhances the growth performance of aquatic animals, a benefit closely linked to the bioactive peptides generated during plant protein hydrolysis. In our previous study, we developed a recombinant *Bacillus subtilis* expressing the P4’ peptide of cottonseed meal (P4’ peptide-bearing *B. subtilis*). We observed that the addition of this recombinant *B. subtilis* at a dose of 10^9^ CFU/kg to the diet of *E. sinensis* produced a significant growth-promoting effect. The mechanism underlying this growth-promoting effect remains unclear. This experiment aimed to investigate the effects of feed supplementation with P4’ peptide-bearing *B. subtilis* on the growth performance, digestive enzyme activities, serum protein content, antioxidant-related enzyme activities, and genes associated with the PI3K/AKT/TOR pathway in the Chinese mitten crab (*Eriocheir sinensis*). A total of 240 crabs (mean body weight: 0.85 ± 0.01) were randomly assigned to three groups and provided with three distinct diets: one without *B. subtilis* (CON), one containing 10^9^ CFU/kg of unmodified *B. subtilis* (BS9), and one with P4’ peptide-bearing *B. subtilis* (RBS9) at 10^9^ CFU/kg. After 8 weeks of feeding, the BS9 and RBS9 groups had significantly increased specific growth rate, average body weight, muscle crude protein content, hemolymph total protein/albumin/globulin levels, hepatopancreatic digestive enzyme (protease and amylase) activities, and *tor* expression in crabs compared to the CON group. Conversely, muscle water content, hepatopancreatic ROS levels, and *4ebp-1* expression were significantly reduced. Furthermore, mean body weight, protein retention, muscle crude fat content, total hemolymphatic protein, and globulin content, as well as the hepatopancreatic expression of *pi3k*, *tor*, and *s6k1*, were significantly higher in the RBS9 group compared to the BS9 group. In contrast, the hepatopancreatic ROS level exhibited a declining trend. The result demonstrated that compared to unmodified *B. subtilis*, incorporating 10^9^ CFU/kg P4’ peptide-bearing *B. subtilis* into the feed significantly enhanced the growth performance and protein deposition in *E. sinensis*. This enhanced effect coincided with the upregulation of key PI3K/AKT/TOR pathway genes (*pi3k*, *tor*, *s6k1*) and the downregulation of *4ebp-1* in the hepatopancreatic cells, indicating a potential role of this pathway in mediating the observed growth promotion.

## Simple Summary

Chinese mitten crab, commonly known as hairy crab, is an important aquaculture species in China, and artificial compound feed to replace trash fish for farming this species often leads to slow growth and affects economic efficiency. In this study, we investigated the effects of P4’ peptide-bearing *Bacillus subtilis* (a modified probiotic expressing cottonseed meal-derived P4’ peptide) on the growth and health of crab by adding it to the feed. It was found that feed supplemented with the strain significantly enhanced the growth rate, muscle protein content, and digestive enzyme activities of crabs while reducing the level of oxidative stress. Further analysis showed that the strain promoted protein synthesis and enhanced immunity by activating the expression of genes related to the PI3K/AKT/TOR signaling pathways. These results provide new ideas for the development of efficient and environmentally friendly aquafeeds, which can help to shorten the culture cycle, increase production, and promote the sustainable utilization of plant protein resources in aquaculture.

## 1. Introduction

The Chinese mitten crab (*Eriocheir sinensis*) is a prominent aquaculture species in China, valued by consumers for its palatable taste and nutritional benefits. The expansion of consumer demand has led to an increase in the scale of *E. sinensis* culture, with annual production reaching approximately 800,000 tons [1]. With the advancement of the feed industry and the enforcement of environmental protection policies, artificial compound feed has increasingly supplanted traditional biological baits, such as chilled fish, in the culture of *E. sinensis*. In contrast with traditional biological baits like chilled fish, *E. sinensis* that are fed artificial compound feed typically exhibit slower growth rates (mainly in terms of molting performance, muscle growth, and gonadal development). This hinders timely marketing, limiting the ability to capitalize on the peak price seasons of the Mid-Autumn Festival and National Day, and ultimately leading to reduced economic benefits compared to crabs fed biological baits like chilled fish. This phenomenon has become a pressing practical issue in the feed-based intensive culture of *E. sinensis*. In the feed industry, plant proteins have become an important protein source in aquafeeds due to their cost-effectiveness and wide range of sources. Among them, cottonseed meal (CM), as one of the major by-products of oilseed processing, boasts huge production and low cost, holding significant potential in aquafeed ingredient development. However, the direct utilization of CM is constrained by issues such as high levels of anti-nutritional factors (such as free gossypol), imbalanced amino acid profile, and poor palatability, limiting its extensive application in aquafeeds. Previous studies indicated that incorporating an appropriate quantity of cottonseed meal protein hydrolysate in place of cottonseed meal (isonitrogenous) into the feed could enhance the growth rate of *E. sinensis*, thereby reducing the market cycle [2,3,4]. The mechanism underlying this growth-promoting effect remains unclear. Notably, the concentration of small peptides (180–1983 Da) rose by 605.26% following the hydrolysis of cottonseed meal protein [5]. Additionally, small peptides have demonstrated growth-promoting effects in aquatic animals, primarily by enhancing nutrient utilization efficiency and modulating key physiological functions related to growth [6,7]. Therefore, we hypothesized that the growth-promoting effects of cottonseed meal protein hydrolysate are closely related to small peptides.

Plant proteins serve as a cost-effective and abundant source in aquafeed, partially or fully replacing fishmeal. Their application progresses through three stages: (1) direct incorporation of crushed oilseed meal by-products; (2) enzymatic hydrolysis of traditional plant proteins under controlled conditions to produce hydrolysates; and (3) analysis of hydrolysate peptide sequences using electrospray ionization–liquid chromatography–tandem mass spectrometry (ESI-LC-MS/MS) [8]. Biologically active peptides were identified for introduction into the *B. subtilis* expression system for mass production, and subsequently incorporated into the feed [9]. In the future, optimizing the fermentation process parameters, determining the appropriate addition amount for different aquatic animals, and comparing the effect with the commercially available replacement anti-antibody products will help to promote the application of plant-derived active peptide-bearing *B. subtilis* in commercial aquatic feeds.

The PI3K/AKT/TOR signaling pathway is a central regulator of cellular growth, metabolism, and protein synthesis in eukaryotes. The activation of this pathway enhances nutrient uptake, promotes anabolic processes, and inhibits catabolism, ultimately driving tissue growth and development [10,11]. In crustaceans, including *E. sinensis*, this pathway is implicated in mediating the effects of nutritional stimuli on growth performance and muscle protein deposition [12,13]. Given that bioactive peptides from plant hydrolysates are known to modulate growth-related signaling cascades in vertebrates [14], we hypothesize that P4’-bearing *B. subtilis* may exert its growth-promoting effects in *E. sinensis* via the PI3K/AKT/TOR pathway. Investigating this mechanism is critical to elucidate how peptide-enhanced feeds accelerate growth in aquaculture species.

A prior study indicated that incorporating P4’ peptide-bearing *B. subtilis* at a concentration of 10^9^ CFU/kg into feed enhanced the growth performance of *E. sinensis* [9]. This finding implies that P4’ peptide-bearing *B. subtilis* may serve as an effective growth promoter for aquatic species. The underlying mechanisms of these beneficial effects remain inadequately understood. This research aimed to evaluate the effects of P4’ peptide-bearing *B. subtilis* on the growth, digestive enzyme activities, serum protein content, antioxidant-related enzyme activities, and genes associated with the PI3K/AKT/TOR pathway in *E. sinensis*. The results will enhance our understanding of the growth-promoting mechanisms of plant protein hydrolysates, facilitate the application of cottonseed meal by-products in aquaculture, and offer new insights into addressing the slow growth of *E. sinensis* when fed artificial compound feeds.

## 2. Materials and Methods

### 2.1. Experimental Methods

In keeping with our previous study [9], unmodified *B. subtilis* and P4’ peptide-bearing *B. subtilis* (both unmodified and recombinant *B. subtilis* are *B. subtilis* JC-66, NCBI accession number: SAMN37990396), both at 10^9^ CFU/kg, were incorporated into the basal feed as experimental feeds, designated as BS9 and RBS9 groups, respectively. The control group (CON) received a basal feed, the formula for which is outlined in Table 1, while the P4’ peptide-bearing *B. subtilis* was prepared following the method described in reference [9]. Protein sources included soybean meal, cottonseed meal, rapeseed meal, peanut meal, fish meal, and blood meal, while fat sources comprised fish oil and soybean oil. α-starch served as the primary carbohydrate source. All feed ingredients were ground using an 80 mesh grinder (Tianjin Taisite Instrument Co., Ltd., Tianjin, China) and subsequently fully mixed using a step-by-step premixing process. Following this, the fat source and distilled water were added sequentially, and the feed ingredients were mixed thoroughly once more. The mixed ingredients were extruded using a single screw extruder (Wenger X-20, Wenger Manufacturing; Sabetha, KS, USA) with a diameter of 1.0 mm, subsequently air dried, and stored at −20 °C.

### 2.2. Management of Crab

All crabs utilized in this experiment were sourced from the Aquaculture Station of Nanjing Agricultural University located in Pukou, Jiangsu, China. The crabs were temporarily housed in a cement pond and fed basal feed twice daily. Two hundred forty healthy individuals (mean initial body weight: 0.85 ± 0.01) were randomly selected after one week and assigned to twelve white buckets (dimensions: 2.0 × 1.2 × 0.5 m), with twenty individuals in each bucket. Each diet was administered at 4–6% of body weight to crabs across four buckets over a duration of eight weeks. Throughout this period, one-third of the water was replaced daily, and residual feed was removed. Water temperature, dissolved oxygen, pH, salinity, and ammonia levels were maintained at 24–28 °C, 5 mg/L, 8.0–8.5, less than 300 mg/L, and less than 0.05 mg/L, respectively.

### 2.3. Sample Collection and Analysis

#### 2.3.1. Sample Collection

Sixteen crabs were randomly selected as initial samples prior to the commencement of the culture trial. At the conclusion of the culture, all experimental crabs were subjected to a 24 h starvation period and subsequently placed on ice packs for cryoanesthesia. Each crab was weighed to facilitate the calculation of growth data. Six crabs were randomly selected from each bucket for sampling, which was performed on ice packs during the process. Hemolymph was initially collected with a sterile syringe containing anticoagulant [15]. The anticoagulant hemolymph mixture was then centrifuged at 3500 rpm for 20 min at 4 °C, and the supernatant was stored at −80 °C for future use. The hepatopancreas was subsequently excised, weighed, and stored at −80 °C for preservation. Claws, legs, and abdominal muscles were excised using scalpels and ophthalmic scissors, subsequently weighed, and stored at −20 °C for preservation.

#### 2.3.2. Proximate Composition Analysis

The moisture content in muscle was determined by drying to a constant weight at 105 °C. The contents of crude protein, crude fat, and crude ash in muscle were determined through Kjeldahl determination, Soxhlet extraction, and direct ashing at 550 °C, respectively.

#### 2.3.3. Assay of Digestive Enzyme Activity

Hepatopancreas samples were collected and homogenized with nine volumes of saline. The homogenized mixture underwent centrifugation at 4 °C for 15 min at 9000 rpm, and the supernatant was collected for enzyme activity assessment. Protease activity was quantified as 1 mg of supernatant producing 1 µg of tyrosine per minute during casein hydrolysis, defined as 1 unit of enzyme activity [16]. Amylase activity was quantified as 1 µg of glucose generated from the hydrolysis of soluble starch per minute per 1 mg of supernatant, representing 1 unit of enzyme activity. Lipase activity was quantified as 1 unit of enzyme activity for the conversion of 1 µg of fatty acids from fat per minute per 1 mg of supernatant.

#### 2.3.4. Hemolymphatic Protein Level

The total hemolymphatic protein content was assessed using the Biuret and BCG dye binding methods [17]. The bromocresol green binding method was utilized to determine hemolymph albumin content [18]. Globulin content was calculated by deducting albumin content from total protein content.

#### 2.3.5. Analysis of ROS Levels

The hepatopancreas tissues of *E. sinensis* were freshly removed and prepared into a cell suspension following the method outlined by reference [19]. The reactive oxygen species (ROS) content was quantified using an ROS assay kit (Jiangsu Kaiji Biotechnology Co., Ltd., Nanjing, China, item number KGT010-1) and following the provided instructions meticulously. The collected cells were resuspended in 2′,7′-dichlorodihydrofluorescein diacetate (DCFH-DA), incubated at 37 °C for 20 min, and mixed up and down every 5 min to ensure complete interaction between the probe and the cells. Intracellular reactive oxygen species oxidized the non-fluorescent DCFH to produce fluorescent dichlorofluorescein. Subsequently, the ROS content was measured using a Beckman Coulter cytometer (Cytoflex, Beckman Coulter, Brea, CA, USA) with an excitation wavelength of 488 nm and an emission wavelength of 530 nm.

#### 2.3.6. Transcriptional Analysis

For transcriptional analysis, 100 mg of hepatopancreas was frozen at −80 °C and transferred to EP tubes. Subsequently, 1 mL of Trizol solution (Sigma, St. Louis, MO, USA) was added for homogenization. After a 10 min incubation in an ice box, 200 µL of chloroform was introduced and mixed thoroughly, and the sample was centrifuged at 12,000× *g* for 10 min. The supernatant was collected, and an equal volume of isopropanol was added. The mixture was allowed to stand for 10 min and subsequently centrifuged at 12,000× *g* for 15 min. Following the removal of the supernatant, ethanol was introduced and mixed thoroughly, after which centrifugation was performed at 12,000× *g* for 5 min. The ethanol was then evaporated, and enzyme-free sterile water was added to dissolve the RNA. The RNA concentration was quantified and subsequently diluted. The diluted RNA underwent reverse transcription to cDNA utilizing a reverse transcription kit (PrimeScript RT Master Mix, Takara, Japan) and was subsequently stored at −20 °C. The expression of target genes was assessed using the SYBR Premix Ex Taq TM II kit (Takara Biotechnology Co., Ltd., Dalian, China). Primer sequences for the target genes are presented in Table 2, and the amplification efficiencies for all primers exceeded 90%. Relative gene expression was determined using the 2^−∆∆CT^ method [20], employing ubiquitin/ribosomal *s27* fusion protein (*s27*) as the internal reference gene [21].

### 2.4. Statistical Analysis

After completing the check for homogeneity and normality of the obtained data, statistical analysis was performed using SPSS (IBM SPSS 25.0, SPSS Ins., Armonk, NY, USA). Independent sample *t*-tests were performed to compare CON and BS9 groups, CON and RBS9 groups, and BS9 and RBS9 groups, respectively. All data were expressed as mean ± S.E.M. A significant difference between the two groups was determined when *p* < 0.05.

## 3. Results

### 3.1. Growth Performance and Muscle Composition

**Figure 1:**
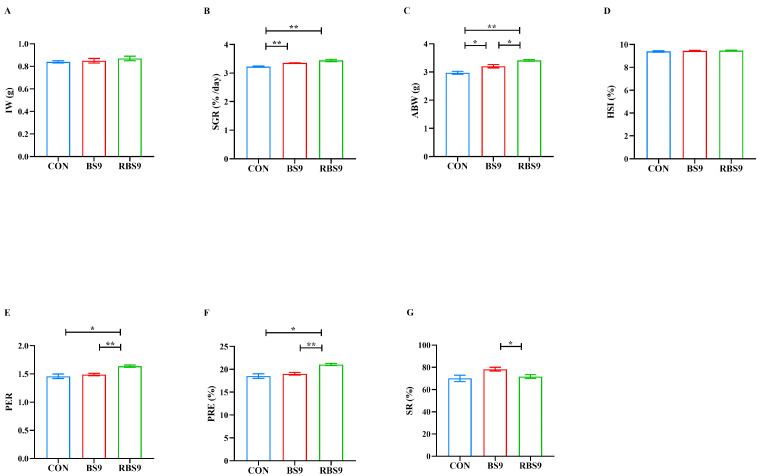
Effect of dietary supplementation with unmodified and recombinant *B. subtilis* on growth performance of *E. sinensis*. (**A**) Initial body weight (IW, g); (**B**) specific growth rate (SGR, %/day) = (Ln final body weight (g) − Ln initial body weight (g)) × 100/culture period (day); (**C**) average body weight (ABW, g) = (initial body weight (g) + final body weight (g))/2; (**D**) hepatopancreas index (HSI, %) = (hepatopancreas weight (g) × 100)/body weight (g); (**E**) protein efficiency ratio (PER) = (final body weight (g) − initial body weight (g)/protein intake (g); (**F**) protein retention efficiency (PRE, %) = 100 × body protein gain (g)/protein intake (g); (**G**) survival rate (SR, %) = 100 × final survival crabs number/initial crabs number. The number of samples is four. Significance is presented as * *p* < 0.05, ** *p* < 0.01.

**Figure 2:**
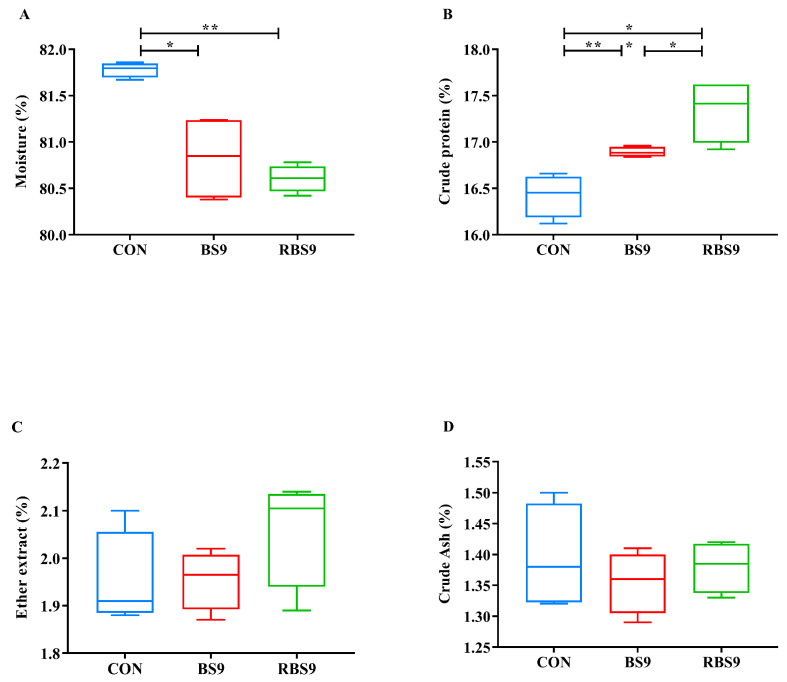
Effect of dietary supplementation with unmodified and recombinant *B. subtilis* on muscle composition (%, wet weight) of *E. sinensis*. (**A**) moisture; (**B**) crude protein; (**C**) ether extract; (**D**) crude ash. The number of samples is six. Significance is presented as * *p* < 0.05, ** *p* < 0.01.

Figure 1 and Figure 2 indicate that there were no significant differences (*p* > 0.05) in initial weight (IW) and hepatopancreatic index (HIS) across the three groups. The specific growth rate (SGR), average body weight (ABW), muscle moisture, and crude protein content were significantly reduced in the CON group compared to the two *B. subtilis* groups (*p* < 0.05). The protein efficiency ratio (PER) and protein retention rate (PRE) of the CON group were significantly lower than those of the RBS9 group (*p* < 0.05), while no significant difference was observed compared to the BS9 group (*p* > 0.05). In the comparison of the two *B. subtilis* groups, the PER, PRE, and muscle crude protein content were significantly reduced in the BS9 group relative to the RBS9 group (*p* < 0.05), while the survival rate (SR) showed the opposite trend (*p* < 0.05).

### 3.2. Activities of Digestion-Related Enzymes

**Figure 3:**
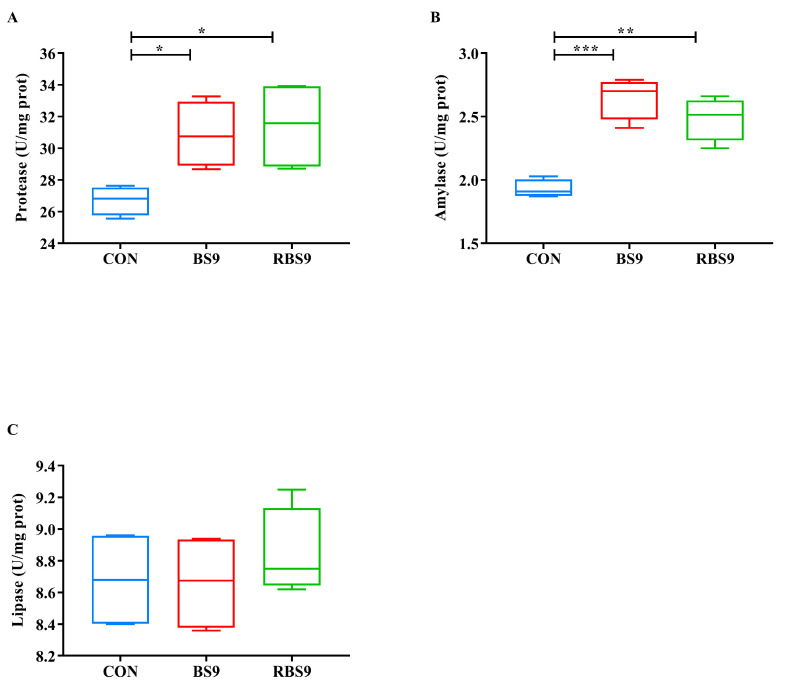
Effect of dietary supplementation with unmodified and recombinant *B. subtilis* on the hepatopancreatic digestive enzyme activities of *E. sinensis*. (**A**) Protease; (**B**) amylase; (**C**) lipase. The number of samples is six. Significance is presented as * *p* < 0.05, ** *p* < 0.01, *** *p* < 0.001.

Figure 3 illustrates that there was no significant difference in lipase activity across the three groups (*p* > 0.05). The hepatopancreatic protease and amylase activities in the CON group were significantly lower than those observed in the two *B. subtilis* groups (*p* < 0.05). However, no significant difference was found between the two *B. subtilis* groups (*p* > 0.05).

### 3.3. Levels of Hemolymphatic Protein

**Figure 4:**
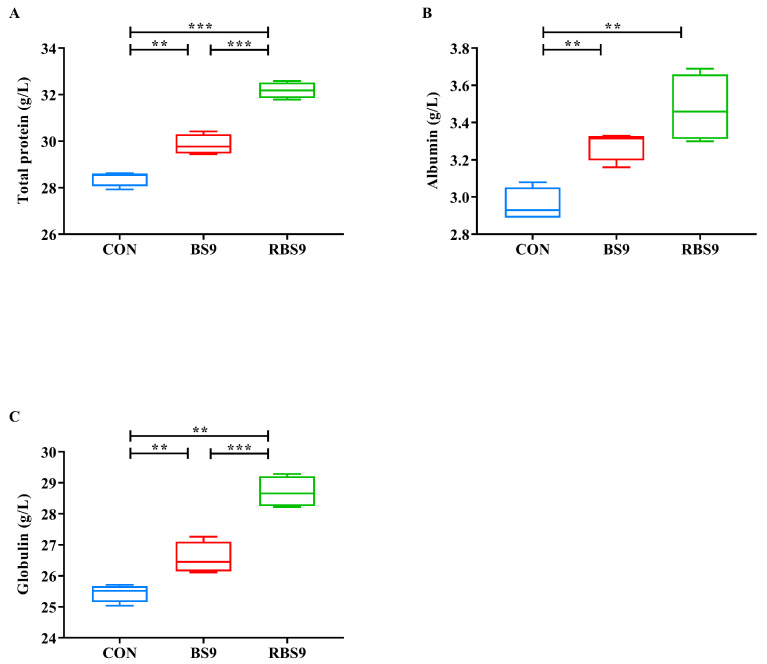
Effect of dietary supplementation with unmodified and recombinant *B. subtilis* on hematolymphatic protein levels of *E. sinensis*. (**A**) Total protein; (**B**) albumin; (**C**) globulin. The number of samples is six. Significance is presented as ** *p* < 0.01, *** *p* < 0.001.

Figure 4 illustrates that the total protein, albumin, and globulin levels in the hemolymph of the CON group were significantly lower than those observed in the two *B. subtilis* groups (*p* < 0.01). In the comparison of the two *B. subtilis* groups, the total protein and globulin content in the BS9 group was significantly lower than that in the RBS9 group (*p* < 0.001). However, no significant difference was observed in albumin content between the two groups (*p* > 0.05).

### 3.4. ROS Levels and Relative Expression of PI3K/AKT/TOR Pathway-Related Genes in the Hepatopancreas

**Figure 5:**
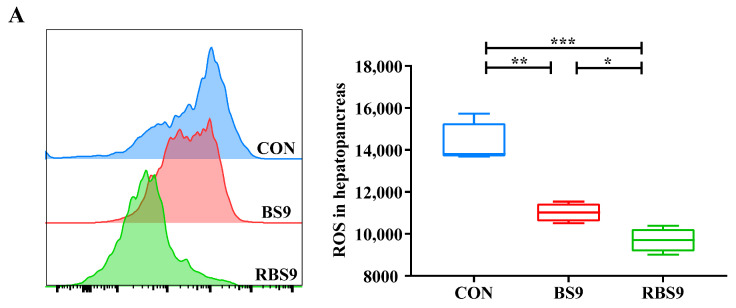

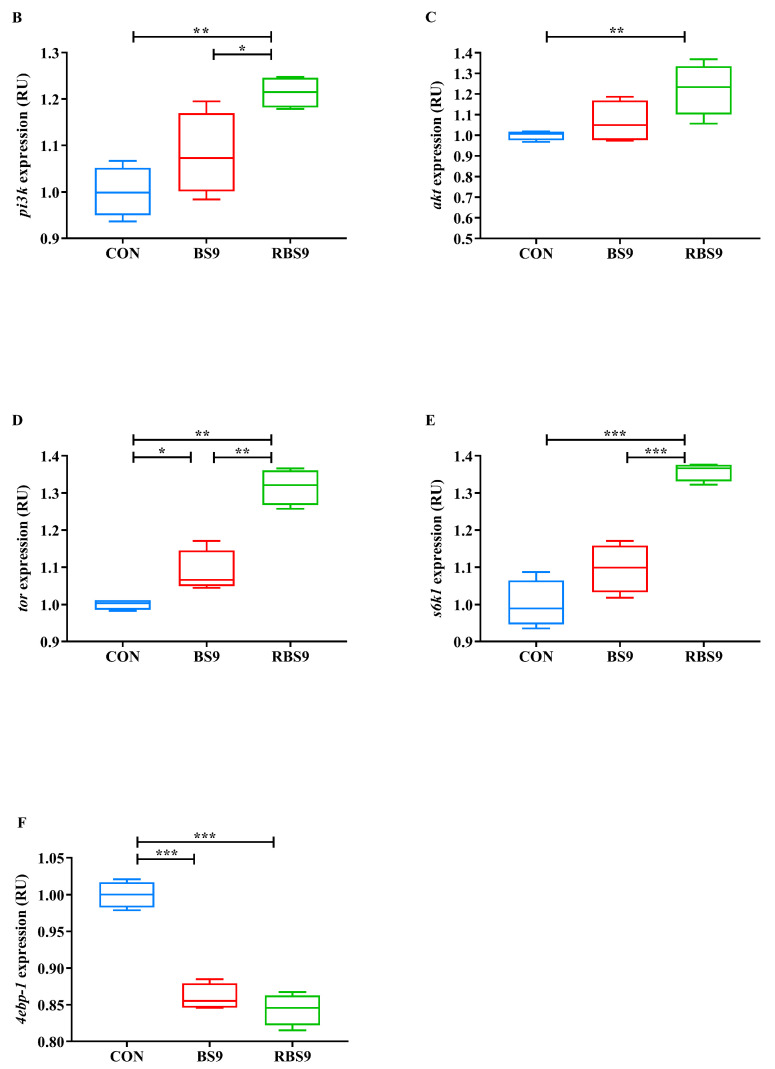
Effect of dietary supplementation with unmodified and recombinant *B. subtilis* on the reactive oxygen species relative fluorescence intensity in hepatopancreas cells (ROS, (**A**)) and the transcription of insulin-like growth factor-1 (*igf-1*, (**B**)), serine/threonine protein kinase B (*akt*, (**C**)), target of rapamycin (*tor*, (**D**)), protein S6 kinase 1 (*s6k1*, (**E**)), and 4E-binding protein 1 (*4ebp-1*, (**F**)) in the hepatopancreas of *E. sinensis*. The number of samples is six. Significance is presented as * *p* < 0.05, ** *p* < 0.01, *** *p* < 0.001.

Figure 5 illustrates that the expression of tor in the CON group was significantly lower compared to the two *B. subtilis* groups (*p* < 0.05). In contrast, the levels of ROS and the expression of *4ebp-1* exhibited a significantly higher opposite trend (*p* < 0.001). In a comparison of the two *B. subtilis* groups, the expression levels of *pi3k*, *tor*, and *s6k1* in the BS9 group were significantly lower than those in the RBS9 group (*p* < 0.05). Conversely, the ROS levels exhibited a significantly higher trend in the BS9 group (*p* < 0.05). The expression levels of *pi3k*, *akt*, and *s6k1* in the CON group were significantly lower than those in the RBS9 group (*p* < 0.01), while no significant difference was observed compared to the BS9 group (*p* > 0.05).

## 4. Discussion

The SGR, ABW, muscle moisture, and crude protein contents of the CON group were significantly lower than those of the two *B. subtilis* groups in this study. This indicates that both unmodified *B. subtilis* and P4’ peptide-bearing *B. subtilis* enhance growth performance and promote organic matter deposition in *E. sinensis*. Previous studies confirm that *B. subtilis* is abundant in proteins, minerals, and vitamins and can secrete various digestive enzymes that enhance nutrient digestion and absorption. The inclusion of *B. subtilis* in aquatic animal feeds in appropriate quantities has the potential to improve feed utilization, nutrient deposition, and growth performance [23]. This study found that the PER and PRE of the CON group were significantly lower than those of the RBS9 group but not significantly different from the BS9 group. In the comparison of the two *B. subtilis* groups, the ABW, PER, PRE, and muscle crude protein content of the BS9 group were significantly lower than those of the RBS9 group. This indicates that *B. subtilis* bearing the P4’ peptide exhibits superior growth performance and protein deposition relative to unmodified *B. subtilis*. The precise mechanism remains ambiguous owing to the limited number of relevant studies. To elucidate the mechanism underlying this beneficial effect, we measured the activities of digestive enzymes. In addition, we observed an interesting phenomenon that the SR of the RBS9 group was significantly lower than that of the BS9 group, but not significantly different from that of the CON group. We hypothesized that the improved molting performance might be one of the reasons for the superior growth performance of P4’ peptide-bearing *B. subtilis* compared to unmodified *B. subtilis* and *E. sinensis*, as a crustacean, whose molting activity is usually accompanied by a certain mortality rate.

Protease, amylase, and lipase are the primary digestive enzymes in the digestive tract of animals, and the organism’s growth is closely linked to the activities of these enzymes. The hepatopancreatic protease and amylase activities in the CON group were significantly lower than those observed in the two *B. subtilis* groups. This suggests that both unmodified *B. subtilis* and *B. subtilis* bearing the P4’ peptide can enhance the organism’s capabilities. This aligns with earlier research on Indian white shrimp (*Fenneropenaeus indicus*), Pacific white shrimp (*Litopenaeus vannamei*), and grass carp (*Ctenopharyngodon idella*) [24,25,26]. *B. subtilis*, as a probiotic, secretes digestive enzymes, including cellulase, protease, and amylase. These enzymes, in conjunction with endogenous enzymes present in the animal’s digestive tract, enhance the activity of digestive enzymes within the organism, thereby facilitating nutrient digestion and absorption. Increased protease and amylase activities in the two *B. subtilis* groups typically signify an enhanced capacity of the organism to acquire the necessary nutrients for growth from feed [27]. This study found no significant difference in hepatopancreatic protease and amylase activities between the two *B. subtilis* groups. This suggests that the P4’ peptide-bearing *B. subtilis* maintains its function of enhancing digestive enzyme activities in aquatic animals post-modification. The findings indicate that the superior growth performance and protein deposition of P4’ peptide-bearing *B. subtilis*, relative to unmodified *B. subtilis* in this study, were not attributable to enhanced activities of the three primary digestive enzymes.

This study found that total hemolymph protein, albumin, and globulin levels were significantly reduced in the CON group compared to the two *B. subtilis* groups. This indicates that *B. subtilis* may contribute to growth promotion by improving the protein metabolism of *E. sinensis*. Proteins in arthropod hemolymph serve to transport signaling molecules and nutrients, regulate osmotic pressure, and activate the immune system [28]. Albumin levels are directly associated with the nutritional status and protein synthesis capacity of the organism [29], whereas globulin levels reflect the inflammatory and immune status of the organism [30]. There is a positive correlation between total protein levels and the growth performance of aquatic animals [31]. The measurement of total protein and albumin levels reflects protein absorption, synthesis, and catabolism in animals [32]. This study observed that total protein and globulin levels were significantly lower in the BS9 group compared to the RBS9 group. This may elucidate the superior growth performance and protein deposition effects of P4’ peptide-bearing *B. subtilis* in comparison to unmodified *B. subtilis*.

The growth-promoting function of P4’ peptide-bearing *B. subtilis* may be attributed to the enhancement of protein metabolism, as indicated by hemolymph protein levels. The PI3K/TOR/AKT pathway is the primary focus in the regulation of protein synthesis and cell growth in protein metabolism [33]. The TOR pathway is activated through the mediation of PI3K and AKT. AKT, a crucial component of the PI3K/AKT pathway, relies on the activation of the upstream PI3K. The classical PI3K/AKT/TOR pathway consists of PI3K, AKT, and TOR [34], with the regulation of protein synthesis being a significant function [35]. Reactive oxygen species (ROS) function as an upstream regulator of the PI3K/AKT/TOR pathway [36], with increased levels of ROS leading to the inhibition of this pathway [37,38,39]. P4 peptide (LGSPDVIVIR) is an active peptide with excellent antioxidant function in vitro that we screened from cottonseed meal protein hydrolysate [8]. It was verified to be stably expressed as a tandem (P4’) of five P4 peptides in P4’ peptide-bearing *B. subtilis* [9]. The P4’ peptide carried by P4’ peptide-bearing *B. subtilis* is cleaved by endogenous trypsin at the arginine site into a single P4 peptide with antioxidant function, which enhances the body’s antioxidant capacity through activation of the SOD-CAT enzymatic antioxidant system [9,40]. As the body’s antioxidant capacity increases, the accumulation of ROS decreases [41], which, in turn, activates the PI3K/AKT/TOR pathway. This study found that the expression of tor in the CON group was significantly lower than in the two *B. subtilis* groups, whereas the levels of ROS and *4ebp-1* expression exhibited an inverse relationship. The expression levels of *pi3k*, *akt*, and *s6k1* in the CON group were significantly lower than those in the RBS9 group but did not differ significantly from the BS9 group. The expression levels of *pi3k*, *tor*, and *s6k1* in the BS9 group were significantly lower compared to those in the RBS9 group. This indicates that the enhanced growth performance and protein deposition effect of P4’ peptide-bearing *B. subtilis*, in comparison to unmodified *B. subtilis*, may result from the activation of the PI3K/AKT/TOR pathway via a reduction in the ROS levels. PI3K is a lipid kinase composed of a catalytic subunit [42]. Its activation produces the lipid second messenger phosphatidylinositol-3,4,5-triphosphate, which activates the downstream target AKT, leading to the transmission of signals to the downstream target TOR. TOR promotes protein synthesis through the phosphorylation of S6K1 and the inhibition of 4EBP-1 expression [4,22,43] across species from yeast to humans [44,45].

## 5. Conclusions

This study demonstrated that incorporating 10^9^ CFU/kg of unmodified *B. subtilis* and P4’ peptide-bearing *B. subtilis* into feed enhanced growth performance, digestive enzyme activities, and hemolymph protein content while decreasing ROS levels in the hepatopancreatic cells of *E. sinensis*. Compared with unmodified *B. subtilis*, P4’ peptide-bearing *B. subtilis* reduced ROS levels in hepatopancreatic cells, which effectively activate key genes of the PI3K/AKT/TOR pathway and efficiently promote protein synthesis. This modification resulted in improved growth performance and protein deposition in *E. sinensis*. This study’s results will enhance the utilization of plant proteins and their by-products in aquafeeds.

## Figures and Tables

**Table 1:** Formulation and proximate composition of the basal feed.

Ingredients (%)	
Fish meal	30.00
Blood meal	4.00
Soybean meal (defatted)	10.00
Cotton meal	4.00
Peanut meal	18.81
Rapeseed meal	2.00
α-starch	20.93
Soybean oil	3.55
Fish oil	1.00
Ca(H_2_PO_4_)_2_	1.50
Zeolite powder	0.9
Premix ^a^	1.00
Mixture ^b^	2.30
Proximate composition (% dry-matter basis)	
Dry matter	89.46
Crude protein	39.78
Crude lipid	7.51
Nitrogen-free extract	27.09
Metabolizable energy (MJ/kg)	10.77
Gross energy (MJ/kg)	17.20

^a^ Premix supplied the following minerals (g/kg) and vitamins (IU or mg/kg) per kg: CuSO_4_·5H_2_O, 2.0 g; FeSO_4_·7H_2_O, 25 g; ZnSO_4_·7H_2_O, 22 g; MnSO_4_·4H_2_O, 7 g; Na_2_SeO_3_, 0.04 g; KI, 0.026 g; CoCl_2_·6H_2_O, 0.1 g; vitamin A, 900,000 IU; vitamin D, 200,000 IU; vitamin E, 4500 mg; vitamin K_3_, 220 mg; vitamin B_1_, 320 mg; vitamin B_2_, 1090 mg; vitamin B_5_, 2000 mg; vitamin B_6_, 500 mg; vitamin B_12_, 1.6 mg; vitamin C, 10,000 mg; pantothenate, 1000 mg; folic acid, 165 mg; choline, 60,000 mg; biotin, 100 mg; *Myo*-inositol 15,000 mg. ^b^ Mixture includes the following ingredients (%): choline chloride 4.21%; antioxidants 1.26%; mildew-proof agent 2.09%; salt 21.03%; lvkangyuan 63.15%; and biostimep 8.26%.

**Table 2:** Nucleotide sequences for real-time PCR primers.

Target Gene	Sequence (5′-3′)	Tm (°C)	AT (°C)	References
*pi3k*	(F) AGCATCATGTTCCCTGCCAA	59.96	56	JX452100.1
	(R) GCAGTTTGCGCTCTTGTTCA	59.97		
*akt*	(F) CAAGATCCTGCGCAAAGACG	59.90	56	KY412800.1
	(R) CATGACGAAGCAGAGACGGT	60.11		
*tor*	(F) GCAGCCCCAAGGAGATGAAA	60.32	56	[2]
	(R) ACAGTCGAAACGCCCTCATC	60.39		
*s6k1*	(F) TCAATAGCGTCGTCATCG	54.70	54	[22]
	(R) CCCTGCGTGTAGTGGTTG	58.03		
*4ebp-1*	(F) GCAACACGCCAACTAAACTC	57.97	54	[22]
	(R) GCGACACCACCTAATATCCA	57.10		
*s27*	(F) GGTCGATGACAATGGCAAGA	58.26	56	HM177456
	(R) CCACAGTACTGGCGGTCAAA	60.25		

*pi3k*, phosphatidylinositol 3-kinase; *akt*, serine/threonine protein kinase B; *tor*, target of rapamycin; *s6k1*, protein S6 kinase 1; *4ebp-1*, 4E-binding protein 1; *s27*, ubiquitin/ribosomal *s27* fusion protein. F, forward; R, reverse; AT, annealing temperature.

## Data Availability

The data of this research can be obtained from the corresponding author upon reasonable request.
